# A pragmatic evaluation of university student experience of remote digital learning during the COVID-19 pandemic, focusing on lessons learned for future practice

**DOI:** 10.1371/journal.pone.0283742

**Published:** 2023-05-04

**Authors:** Menna Brown, Alice E. Hoon, Maisie Edwards, Shawn Shabu, Imannuella Okoronkwo, Philip M. Newton

**Affiliations:** Swansea University Medical School, Faculty of Medicine, Health and Life Sciences. Singleton Park, Swansea, United Kingdom; Sheffield Hallam University, UNITED KINGDOM

## Abstract

The Covid-19 pandemic and subsequent national lockdowns resulted in drastic changes to the way that higher education was delivered. A mixed-methods research study was conducted to explore university students’ perceptions of online learning during the 2020/21 academic year. Students from across all Welsh higher education institutions were invited to participate. First, a series of focus groups (n = 13) were conducted to explore students’ experiences of online learning during the pandemic. Two were conducted in Welsh, the remaining eleven in English. Thematic analysis led researchers to develop eight key themes: Seeking the positives, Facilitators to learning, Barriers to learning, Lost sense of community, Let down by University, Workload, Assessment, and Health and well-being. These themes informed the design of a quantitative survey which was completed by 759 students. It was found that students were largely satisfied with the quality of online learning, however there were specific challenges associated with a lack of community, wellbeing concerns, and challenges with loneliness and isolation. Data from the focus groups and survey informed recommendations for practice in three key categories; teaching practice, institutional level recommendations, and student health and wellbeing considerations.

## Introduction

The COVID-19 pandemic caused an unprecedented global disruption to everyday life [1], including sudden closures of educational institutions [2]. This included Higher Education Institutions (HEIs) across Wales, United Kingdom which made an immediate and unplanned move to a fully digital, remote, delivery of all teaching and learning in the 20/21 academic year and a blended approach in 21/22. This was a huge challenge for us all. But crisis is often a significant driver of innovation [3], and, even early in the pandemic, there was an expectation that it would drive innovation and long lasting change in Higher Education [4, 5].

Survey studies were deployed across the sector to capture the student experience of this transition to online learning, and the early indications were that the transition to online learning was associated with many negative experiences for students. For example, students reported dissatisfaction with a lack of peer and teacher interaction, and the challenges of engaging exclusively via a screen [6]. Motivation and focus decreased, while students were more distractable [7]. Students reported anxiety and uncertainty about education [8–11], as well as burnout, with cynicism, emotional and mental exhaustion [12, 13]. Overall levels of stress increased, along with a decrease in attention span [14]. Students sense of belonging decreased [11], and their academic social networks fractured, or did not develop at all [10]. Engagement also decreased, an effect that was exacerbated in under-represented groups, in part due to challenges with the home environment [7], and in countries where the technological infrastructure and financial challenges already put students at a disadvantage with online learning [15]. Technical challenges were also reported, including problems with Wi-Fi, and access to suitable quiet space for studying and engaging with online teaching [13]. The lack of access to hands on teaching and laboratory/practical work was a common theme of many of these studies. The studies cited above are from all over the globe, representing every continent.

The transition was also a considerable challenge for university staff, many of whom were not experienced in online teaching and who were dealing with their own challenges caused by the pandemic. This resulted in a significant negative impact of staff wellbeing, increases in workload, and a further erosion of trust in university leadership [16], although studies in the United Kingdom and Pakistan reported that staff felt prepared, supported and competent to engage in online teaching [17, 18]. Where in-person teaching did happen, staff had to familiarise themselves with public health policy, becoming adept at sanitizing and designing socially distanced learning and teaching environments [19].

Universities also experienced significant institutional challenges. There was a need to rapidly train both staff and students so that they could teach and learn online [20]. Different leadership skills were called for, with an emphasis on responsible, experienced, and adaptable leaders [5]. Some of the long established challenges in university leadership, such as the lack of meaningful gender representation, were exacerbated when attempting to tackle pandemic-induced lockdowns which themselves had differential effects on groups with caring responsibilities [21]. There was a variety in institutional responses, for example where resource-rich universities with high student numbers were quicker to use social media to communicate with their students about issues surrounding the pandemic [22].

Despite these challenges, a number of positives also emerged from the data. Students reported enthusiasm about the flexibility and convenience afforded by online learning [8]. A ‘Remote Teaching Satisfaction Scale’ developed to specifically measure student satisfaction with the transition showed that students appreciated the efforts of staff to established a respectful and cordial atmosphere in the online teaching environment [23]. Staff and students were plunged into a communal challenge, which for some, fostered increased collaboration and a sense of community often found during crisis [5, 19].

Overall though, the experience appears to have been a net negative for both staff and students. However, one theme that is common to almost all the research cited here, is a feeling that the pandemic has forced changes to HE that will persist for a long time. For example, an increased proficiency in online delivery may reframe student’s choice about whether or not to actually attend a physical campus, particularly those students from deprived or rural backgrounds where the logistics are especially complicated [24]. Therefore, it is important to try and capture those aspects of online learning and teaching that were effective, and to try and improve those that were ineffective, for future consideration.

In this study we adopted a pragmatic approach to try and capture the student voice through focus groups and a survey. Pragmatism is an approach to education research, that prioritises asking research questions whose answers are intended to be practically useful. In pragmatic research, epistemological and ontological considerations are of less significance to the methodology than the practical utility of the findings [25]. The aim of this study then was explicitly to generate recommendations for future practice in learning and teaching, based upon a pragmatic evaluation of the student experience of remote digital learning at Welsh Universities during the 2020/21 academic year (September 20-July 21). The vast majority of learning and teaching was remote online at this time, with some exceptions for courses in the health professions.

## Method

### Ethics

Ethical approval was granted by Swansea University Medical School Research Ethics committee, stage 1 (reference number 2021–0037 26.4.21) and stage 2 (reference number 2021–0037). All participants provided written consent.

### Participants

A two-staged mixed methods research study was undertaken between March and September 2021. Students registered to study in any Welsh HEI were eligible to participate. There are eight HEIs in Wales, with a total of 81,670 registered undergraduate students (2019/20).

Inclusion criteria for participating in either stage were:

1. A student at a Welsh HEI
2. Aged 18 plus
3. Ability to consent
4. Access to a WiFi/internet enabled device and (freely accessible) zoom software (for focus groups).

### Procedure: Stage 1 qualitative enquiry

Participants were invited to take part in a series of focus group discussions facilitated via Zoom between April and June 2021. The study advert and flyer (S1–S4 Files) were emailed to potential participants via several routes: relevant staff contacts at each HEI, the National Union of Students Wales (NUS), and HEI Students Union (SU) leads, as well as staff who are members of the All-Wales Learning and Teaching Network (LTN), a group set up to oversee sector management and recovery from the COVID-19 pandemic. The study’s digital advert and flyer were posted on relevant student intranet pages or e-mailed to students. Social media pages were established for participant recruitment purposes.

Interested participants provided consent and arranged a pseudonym prior to participation (if requested). Focus groups were facilitated in English and Welsh and video recorded via zoom software. Transcripts were anonymised prior to analysis. Participants were thanked with a £20 voucher.

### Procedure stage 2: All-Wales survey

The questionnaire was hosted online on Survey Monkey™ (the complete survey is given in S1 File) Participant recruitment was the same as for Stage 1. A voluntary prize draw was included to incentivise participation. All data was collected via Survey Monkey™. The survey was live from 15^th^ June 2021 to 30^th^ August 2021. On clicking the survey link, participants were first required to read the Participant Information Sheet. The survey then took them to a Consent page, and once consent had been given, the survey commenced. At the end of the survey, participants could click a second survey link to enter their e-mail address for the optional prize draw. These survey links were kept separately to ensure that it was not possible to link a participants e-mail address to their survey responses, therefore ensuring anonymity. There were 57 questions in the survey (including demographics), under the following sub-headings: About me, Quality of online learning, Community and collaboration, My workload, Online assessments, Accessing teaching and resources, Managing my health when learning online, Caring for dependents, and Thinking ahead. Questions were a mix of closed Likert questions and open, free text questions.

### Data analysis

#### Stage 1

Qualitative data were analysed using interpretative thematic analysis (ITA) informed by the work of Braun and Clarke [26, 27]. A staged process was undertaken [1] familiarisation [2] initial coding was undertaken in the following way: the facilitators (MB, ME, SS, IO) independently read, re-read and coded focus group one and two. Codes identified independently were shared and discussed and compiled into a coding structure document. Agreed codes were then applied to the remaining transcripts. New codes were shared and discussed as they emerged [3]. The final codes were independently organised into themes. Themes were discussed until agreement was reached. Subthemes were created during this iterative process. [4] Finally, theme names were given, and extracts identified for each theme. A final review of each theme was undertaken to ensure no data were missed and coded data aligned with themes. Final themes were discussed with the research team and informed the survey design.

#### Stage 2 –Quantitative survey data

Quantitative survey data were analysed using descriptive statistics and visual analysis of graphed data. Likert-scale data were analysed by one-sample Wilcoxon test to determine whether the distribution of responses was significantly different to the mid-point of the scale. Statistical analysis and graphing were undertaken using Graphpad Prism 9 (San Diego, CA, USA).

#### Stage 2—Qualitative survey data

Qualitative data from each free text question were analysed using a pragmatic content analysis [28, 29], informed by the results from Stage 1 and Stage 2. This was conducted by the research team (AH, MB, PN). Frequency analysis was then undertaken to identify the most commonly occurring themes.

## Results

### Stage 1

#### Participant characteristics

One hundred and twenty-eight expressions of interest from seven different Welsh HEIs were received. A total of 54 participants took part in 13 focus group discussions, two of which were conducted in Welsh. The majority of participants were women (32/54, 59%). Mean age was 24 years old (range 18–64, SD, 9.497), (S2 File). Participants studied a range of 40 different programmes, across all levels of study.

#### Focus group theme

Each focus group included between two and five participants. Duration ranged from 1 hour 13 minutes to 2 hours 18 minutes.

Through qualitative data analysis, the following eight themes were identified: *Seeking the positives*, *Facilitators to learning*, *Barriers to learning*, *Lost sense of community*, *Let down by University*, *Workload*, *Assessment*, *and Health and well-being*. The themes are summarized in Table 1. Overall, the themes describe the wide range of student experiences, both positive and negative, which arose as a result of the pandemic. The themes can inform future teaching and learning by identifying best practice through a better understanding of the context and nuisances of remote, digital and hybrid learning models that students experienced.

**Table 1:** Focus group themes and sub-themes.

Theme name	Sub-themes
*Seeking the positives*	-
*Facilitators to learning*	Sub-theme 1: Interaction and engagement
	Sub-theme 2: Flexibility and control
	Sub-theme 3: Positive communication
*Barriers to learning*	Sub-theme 1: Lost learning opportunities
	Sub-theme 2: technological barriers
	Sub-theme 3: Learning environment
	Sub-theme 4: Poor communication
*Lost sense of community*	-
*Let down by University*	-
*Workload*	-
*Assessment*	-
*Health and well-being*	-

Each theme is outlined briefly below, with participant extracts.

#### Theme 1. Seeking the positives

Participants identified new learning opportunities encountered as a result of changes to the educational environment. These opportunities included learning new software and hardware which led to the development of new technical skills. Participants took time to learn or revise their digital skills and develop their confidence and digital literacy. Participating in extracurricular educational opportunities (e.g., webinars, Instagram takeovers, ambassador roles, internships) and increased access to online conferences helped develop transferable skills. Students also reported that staff created networking opportunities. Participants reflected on what they gained from remote delivery of teaching, for example, inclusion of international voices across time zones, additional learning resources that had not previously been made available, reduced travel, ability to revisit learning materials, laboratory and practical skills being taught in a different way.

*“I’ve had to do a project on Zoom, which was a complete online production of Shakespeare’s The Tempest on Zoom, which was weird, but really good*. *So, it was just another learning experience for me, so it was positives and negatives to what I’m missing out on, and also the new experience that I’m gaining through all this technological voodoo stuff*. *So, you know, it’s branching out, learning new things and taking experience where you can”*. *(Man, FG1, 83–88)*

*“doing more things over Zoom has developed my IT skills because I can, like, understand how to go on Zoom and stuff and, I don’t know, do different stuff*. *So it has developed that element of things that maybe I wouldn’t have done” (Man, FG9, 938–944)*

However, the way such experiences were discussed indicated that participants were searching for a positive outcome during the crisis period, and there were attempts to ‘make the best of a difficult situation’. Positive experiences were countered by comments expressing a desire to return to face-to-face teaching and learning, or a hope for hybrid environments in which peer and staff interaction are reinstated.

#### Theme 2: Facilitators to learning

This theme referred to aspects of remote and digital learning which supported student’s ability to learn. This theme is organised into three sub-themes.

*Sub-theme 1*: *Interaction and engagement*. Interactive teaching methods and approaches which encouraged students to take part and contribute to live sessions aided ability to engage with teaching and learning in both synchronous (live) and asynchronous (pre-recorded) delivery modes.

*“multiple question tests at the end of each week, and it wasn’t for a mark, but still you can catch up and see what is going on, if you can understand the theory or not*. *So, that was really helpful”*. *(Woman, FG1, 418–420)*

*“some modules I’ve got*, *the tutor does a poll halfway through and gets involved, gets us discussing it” (Man, FG4, 1230–1231)*

*Sub-theme 2*: *Flexibility and control*. Remote learning afforded students increased personal control over the time and pace of their learning which helped some organise their time more effectively. For example, some participants explored how they had scheduled their own learning timetables around their home/personal/work and general life commitments. This flexibility was facilitated by the reliance on pre-recorded lectures, the removal of travel requirements and other life commitments (as a result of UK wide lockdown restrictions). Students highlighted that the flexibility and control afforded to them meant that they were better able to take part in learning at times which suited them.

*“I think the most valuable thing in the whole digital approach to learning this year was the flexibility and the availability of the learning*” *(Woman, FG12, 46–50)*

This was particularly prominent for those with pre-existing physical and mental health conditions, whereby health conditions had previously limited or affected their ability to participate in in-person teaching due to fatigue, illness, medical appointments or social anxiety.

*“I’ve got three children, I’ve got a fulltime job, and just that kind of family friendly–and being more flexible*. *I can do it from home while my daughter’s doing whatever she’s doing, you know*. *I think actually a positive from this move is going to be, moving forward, diversity and inclusivity and accessibility for people, and I think that can only be a good thing, you know, a really good thing, and actually a rationale for keeping an element of a blended approach”*. *(Woman, FG10, 150–156)*

Equally, international students highlighted the benefits of technological functions like, pause, speed control and subtitles which helped them better understand learning materials. For others availability of recordings supported active listening, in the knowledge that materials can be re-watched and repeatedly accessed.

*“they’ve been really useful*, *and I go back and like re-watch them constantly” (Woman, FG1, 148–149)*

*Sub-theme 3*: *Positive communication*. Effective and timely communication supported learning and ability to engage, while frequent and supportive communication from lecturers, academic mentors and personal tutors provided support.

*“my academic tutor*, *even though it’s just fifteen minutes*, *is always like, “Oh, how are you?” and like really checks in, which is really nice to have (Woman, FG1, 1036–39)*

#### Theme 3: Barriers to learning

This theme is described via four sub-themes which highlight the range of experiences that had a negative impact on learning and ability to engage in learning.

*Sub-theme 1*: *Lost learning opportunities*. Students identified a range of lost learning opportunities, these included, changes to programme delivery and the structure and format of modules available, the removal of course components such as laboratory experiments, fieldwork, practical workshops, and industry/workplace placements. Participants widely acknowledged their dismay and perceived this to be a barrier to learning which reduced their ability to develop required knowledge and transferable skills. This was particularly prominent for those undertaking scientific, healthcare, education and performing arts degrees.

*“Regards with online labs*, *I mean, it’s just ridiculous*. *I’m doing medical biochemistry*. *If I wanted to go into a research field and work in a lab, dragging a pipette with my mouse into a test tube is completely different compared to physically doing it*. *There’s so many more human errors*. *So, in that aspect, it’s ridiculous, but at the same time of course I understand the situation (Man, FG6, 1068–1072)*

*“We were meant to have a language clinic every Monday, and that was supposed to be for two hours, but we missed most”*. *(Man, FG9, 464–470)*

*Sub-theme 2*: *Technological barriers*. A lack of access to technology (hardware and software), internet enabled devices and limited, poor or intermittent Wi-Fi presented a practical barrier to learning. Particularly evident for those living in shared accommodation where several students were required to negotiate access to Wi-Fi simultaneously or following a return to family homes where other members of the household were also using the Wi-Fi for their own studies or employment purposes. Instances were discussed where students had to rely on expensive 4G data to access live sessions or to avoid experiencing anxiety and worry arising from poor Wi-Fi. This was also a prominent issue for those in rural locations.

*“I’m always like to my housemates, “Can no one use the wi-fi for a bit?” And then I just have to plug in my headphones and my microphone, and just hope that that works, but it is quite worrying that it is all very temperamental, and even if you have good wi-fi, there are always going to be bad days where it could drop out”*. *(Woman, FG1, 968–971)*

Other technology related barriers included limited skill set, confidence and willingness to adapt to technology led learning environments. Further complicated by technical difficulties, poor functionality, inadequate equipment, poor usability and navigation issues of Virtual Learning Environments (VLE) (e.g., different layouts across modules, a general lack of consistency, or poor organisation by individual lecturers), and digital tools which culminated in wasted time, frustration and/or inability to access required learning resources.

*“I would say basic structure–basic infrastructure or a layout*, *just to be sure that everything that will give you points, and ultimately credits*, *is in something called assessment and feedback*, *or assessment and practicals… another teacher would do it differently” (Man, FG2, 232–234 / 237)*

*Sub-theme 3*: *Learning environment*. Participants described how their personal ‘learning environment’ often acted as a barrier to engagement. For example, many had to share IT devices with family (during the UK lockdown), experienced frequent interruptions by family/friends meant that study time wasn’t always available as planned and reduced ability to concentrate. Often participants needed to adapt their study routines and to access content at unusual hours while others did not have a private/safe place to study at all. Others felt embarrassed by their ‘learning environments’ which contributed to poor engagement in live remote teaching, and meant that cameras were not turned on which affected peer engagement and motivation.

*“I know a lot of people are hindered with disturbances*. *I’m quite lucky to be able to just say to my family, “Can I have a bit of quiet on the upper floor for my lecture?” It hasn’t stopped my mother coming in with a hoover when I’m in the middle of a presentation” (Man, FG7, 847–50)*

*“I know that there’s a few on my course who are embarrassed about their rooms and embarrassed about–you know, they don’t want people judging their rooms.”* *She said, “And there’s a few that just simply won’t go on screen unless they’ve got a full face of makeup on, because they just feel that they need to be ready, you know, eyelashes…” (Woman, FG10, 193)*

*Sub-theme 4*: *Poor communication*. Poor communication relating to timetables (last minute timetable changes for lecturers, seminars, meetings and assessment deadlines, poor and/or inconsistent communication including inconsistent use of channels of communication i.e., the message failed to reach students equitably, ad-hoc communication) meant that some students missed out on vital information. This was of particular concern following periods of unexpected illness; and poor and inconsistent communication regarding module content (such as availability of pre-recorded content).

*“we received email at 10pm the day before, actually, now it’s from three till five*. *Have a good night.…* *So I would say that the slightest mistake cost you a lot*. *You miss one email, you miss one notification, you click mark as read or ignore or letter…*. *And you could be losing*. *It’s a dangerous game” (Man, FG2, 190–199)*

#### Theme 4: Lost sense of community

Participants described a real sense of lost community during online learning. In the main this resulted from the loss of all in-person interactions (peer to peer and peer to staff due to UK wide lockdown) and the resultant loss of the hidden curriculum, that is, the benefits associated with learning from and supporting each other academically and socially (including loss of support, passion, energy from others, engagement in wider learning, diverse ways to embed skills).

*“throughout my last two years, I’ve not actually known who are in my class because they just come up with little icons, so that’s why I’m really disappointed”*. *(Man, FG4, 146–148)*

*“you feel still quite alone because you are one of a 100 person–people, and you are alone in this group”*. *(Woman, FG2, 816)*

The lost sense of community had a detrimental impact on student mental health and wellbeing. Many talked about how their experiences of isolation and loneliness led to feelings of unwanted stress, anxiety and depression. Many shared their lowest points, their concerns moving forwards and their hopes for the future (see theme 2). There was a lack of opportunity to meet new people, make friends, form a social group and generally to find a place at university for themselves. The impact was significant, particularly for first year students who were only beginning to embed themselves in university life, form friendships and networks and establish themselves as independent learners.

*“this isn’t emotional, this is a screen, this is a computer, it’s like there’s no one here*. *So yeah, the university experience has not been with humans” (Man, FG4, 497–499)*

However, this sense of loss was not restricted to new students. Participants across all levels of study talked at length about how they missed social interaction arising naturally via in-person interactions in a range of academic and social environments and the how this affected their learning and motivation to learn. For example, the sense of isolation and lack of community meant, for some difficulty engaging with lecture materials, exacerbated further by pre-recorded lecture content which built up over quickly over the semester and became overwhelming. Participants also noted the impact of zoom/screen fatigue, distractions and the monotony associated with studying in one single environment (mainly their university bedroom) with no access to wider university learning environments (e.g., libraries and study halls) which impacted on motivation.

*“The motivation, trying to get motivation behind–because being a student behind a screen is blooming hard work”*. *(Woman, FG13, 1741)*

*“I need to get up and get out of my bedroom*. *I can’t work in my bedroom from my bed*. *That’s where I sleep, not where I work.”* *(Man, FG4, 492–499)*

#### Theme 5: Let down by university

Participants described being let down by the university (and for some the UK or Welsh governments) during the pandemic. Key issues included a lack of access to critical university resources and services which supported learning (including self-directed study) and independent living. For example, library and computer lab closures, removal of transport links (which impaired ability to attend teaching or move about the city), campus-based food halls and food supplies were closed (which meant international students were particularly affected and those isolating). Participants felt there was inadequate provision of mental health resources or support systems for students (particularly Welsh language students where resources were not translated). Fees and monetary issues not addressed by the university which affected individual’s ability to access remote learning resources, and gave way to resentment and anger for those unable to afford technologies required (previously provided via computer labs and libraries). Limited consideration of training needs for themselves and staff. Students discussed examples of staff who were unable to effectively use new technology, who failed to adequately adapt their learning content for remote delivery and who wasted time during live virtual sessions through a lack of understanding of how to use the new technologies in situ. Combined, students felt a significant loss of the anticipated university experiences and important social and cultural rites of passage.

*“if anyone sort of said to me, “What is the one thing that you feel you’ve missed out on?” That would be it, just sort of going into the library and, yeah, like chatting to the staff and things like that, and asking them, you know, “Have you got any books on this?” ‘Cos I think, yeah, although I’m quite good with technology, I did sort of really struggle initially to use like Met Search and stuff … I do kind of miss that–just being in the library.”* *(Woman, FG10, 617–624)*

*“with mental health there’s been no support offered by the University…*. *institutionally, I think there’s been systemic failure”*. *(Man, FG5, 191–201)*

Two focus groups were held through the medium of Welsh. Welsh speaking students faced additional challenges, from a lack of Welsh language mental health support, a dearth of Welsh language provision for digital resources, to practical barriers of rural life with poor Wi-Fi connectivity.

*“finding resources or sources has been a bit harder, especially in Welsh*. *Like, if you need an old book, the chances are that there isn’t an electronic copy available, so you then have to go through the process of ordering the book from the library, see if they release it to you and then, obviously, go and pick that up*. *You’re okay if you live in [redacted], but when I was home, it was a bit more awkward*. *I had to plan a day so that I could pick the books up because the resources just aren’t really available*.” *(Woman, FG9, 592–598)*

#### Theme 6: Workload

Some participants perceived an increase in workload as a result of the increased time and energy required to adapt to new ways of learning, such as, using VLEs, learning new software, and accessing online resources. Participants reported that watching pre-recorded lectures was more time consuming than a traditional in-person lecture, as they required pausing materials, stopping for breaks, distractions, or to follow up on areas of interest. Students also found they would rewind and re-watch the pre-records to ensure understanding and language translation. The incessantness of the digital environments was challenging. There was also a need to develop new skills whilst simultaneously trying to keep up with the teaching content and assessments.

*“uni’s non-stop*. *Because we’ve got Canvas on our phones then, so that’s what we do our work through*. *And that combined with all the announcements I get on that and the WhatsApp group and everything, it’s just–there is no break from uni*. *Your life is now university” (Man, FG5, 596–99)*

#### Theme 7: Assessment

Experiences of online assessment were coupled with perceived higher workloads. The move to open-book examinations led to a reliance on Google™ and a change in the way students revised and studied. Some students noted that they found open-book exams harder and required additional time to prepare and complete the assessments. Others were concerned with technical failures and errors which affected time available to complete assessments. These were exacerbated by varying home learning environments which meant some individuals were disadvantaged.

*“my discipline is only decreasing …*, *I’ve found it that they’ve styled the question that I’m going to still need to look up the answer on Google*, *whether I’m confident or not, just because I can, and if I can, why shouldn’t I?”* *(Man, FG6, 1062–68)*

#### Theme 8: Health and wellbeing

Remote learning impacted on students’ health and well-being. Students discussed their emotional responses to the challenges, changes and adaptations required of them and the support they received or wished they had received. Physical health was impacted by extended period of screen time, inadequate or a complete failure to schedule breaks between timetabled lectures. hidden health conditions were exacerbated by long periods of sedentary screen time, back pain, stiffness, and reduced ability to undertake self-care routines, whereas previously the educational setting had afforded opportunity to move, stretch, and take a break from screens and intense periods of concentration. Mental health was impacted via a lost sense of community and isolation (see theme 4).

*“Tuesdays last term I had lectures from about 9am or 9:30*, *all the way until 6pm. And the one until 6pm always overran because it was a practical*. *So, there’s no time to have lunch*. *I don’t know how we were expected to do that because every lecture ran until the end”*. *(Man, FG8, 833–6)*

*“after a year of being out of social circles, how are we going to fit in there again? How are we going to find our feet in our groups again? Maybe it’s going to be a new group, maybe it’s the same group, but, you know, lots of people will lose that thing that makes them fit into their group*. *So, I think it’s going to be difficult to fit in”*. *(Woman, FG12 420–4)*

The qualitative themes discussed above directly informed the development of the survey for Part 2. Through triangulation and discussion amongst the research team, the themes were translated into distinct areas of enquiry which were used to structure the survey and develop the questions. Four of the qualitative themes were directly translated into survey subsection (workload, assessment, health and wellbeing and lost sense of community). Three of the learning related qualitative themes (*Seeking the positives*, *Facilitators to learning*, *Barriers to learning)* were streamlined to focus on specific areas of learning (Quality of online teaching’ and ‘Accessing teaching and resources’). The final two survey subsections were included to identify the different situations that students noted impacted their ability to study and engage effectively (‘Caring for dependents’) and finally to reflect their hopes and views for the future of remote learning (‘Thinking ahead’).

### Stage 2 results

#### Participant demographics

In total, 759 participants (466 women, 188 men, 13 non-binary, 92 did not want to answer) participated in the survey. Participants were representative of all years of study, from those studying a foundation year, through to those who had been studying for 6–7 years, and came from a range of different degree types. Just over five per cent were foundation students, 72.15% were undergraduate students and 22.67% were postgraduate students. The full range of demographics is shown in S3 File.

#### Quality of online learning

**Fig 1:**
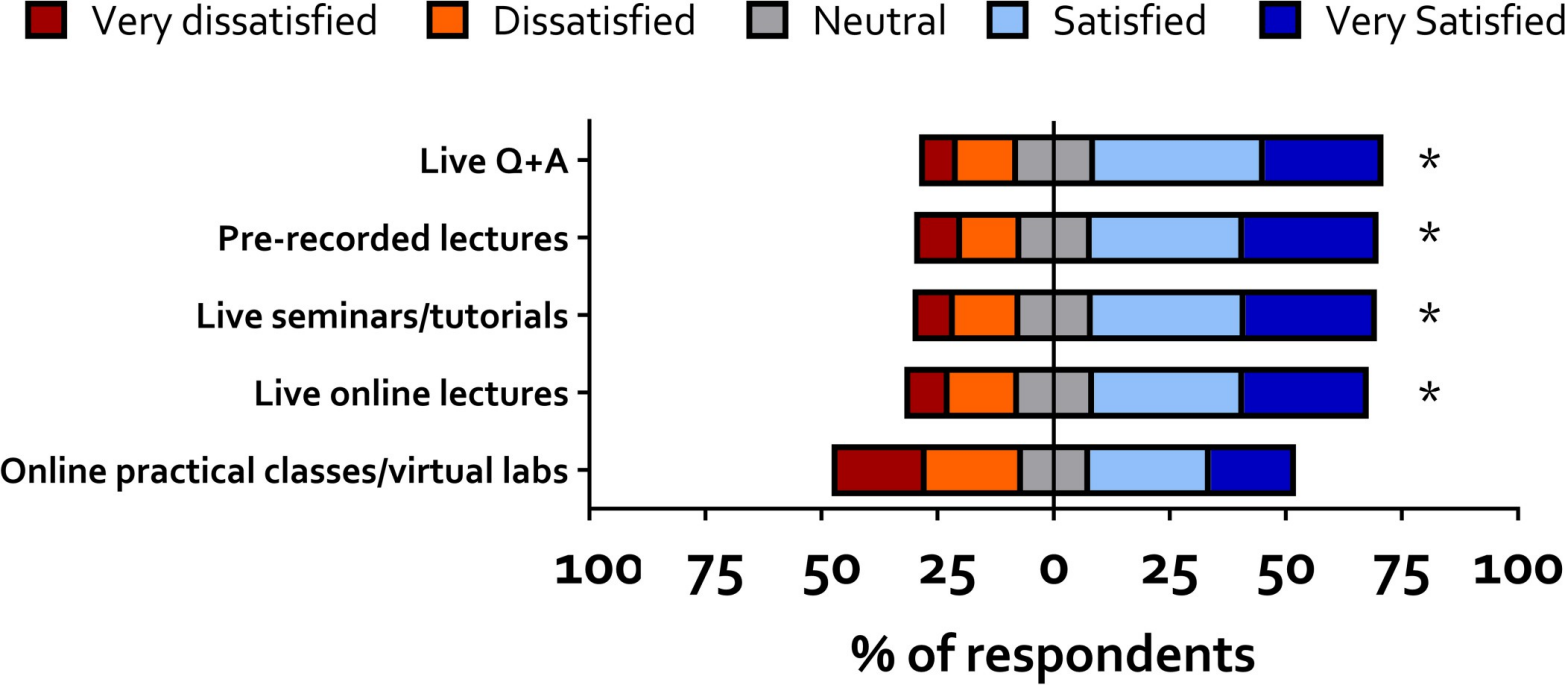
Student satisfaction with different online learning formats. Data shown are arranged around the mid-point of a five-point scale. Distribution of responses was tested using a one sample Wilcoxon signed-rank test to determine whether they were significantly different from the midpoint of the range * = p<0.05. An asterisk to the right of the bar indicates the sample agreed with the statement. No asterisk means the sample neither agreed or disagreed. The distribution of responses indicated that participants were overall satisfied with their experience of most online teaching formats.

Students were asked to rate their satisfaction with their experience of the different types of online learning during the 2020–2021 academic years. Results are shown in Fig 1. For analysis, the five text points on the scale were converted to a numerical 1 (very dissatisfied) to 5 (very satisfied) scale. A one-sample Wilcoxon test was used to test whether, for each online learning format the distribution of responses was significantly different from the midpoint of the scale. The distribution of responses was significantly different for live online lectures (W = 60822, P<0.0001), pre-recorded lectures (W = 53532, P<0.0001), live seminars and tutorials (W = 61135, P<0.0001) and live Q+A (W = 52568, P<0.0001). The distribution was not significantly different for live practical classes and virtual labs (W = 1378, P = 0.75).

These data suggest that, overall, the majority of participants were satisfied with the quality of online teaching. Content analysis of free text data (see S4 File) found that participants would have liked more interactive elements in both live and pre-recorded lectures. Participants had mixed views about how satisfied they were with online practical classes and virtual labs, and free text data suggested that these worked better when recorded in a laboratory environment, and when students were given practical tasks to do.

On the basis of the Stage 1 data, we asked two separate specific questions about breakout rooms used in online teaching; students were asked to state, on a five-point scale, whether they found breakout rooms engaging or useful. For analysis, the five text points on the scale were converted to a numerical 1 (‘Not at all engaging/useful’) to 5 (‘Very engaging/useful’) scale. The majority of participants responded that breakout rooms were not very engaging, with 32.28% selecting that they were ‘Not at all engaging’ and 22.83% selecting ‘A little engaging’ (22.83%). The majority also did not find them very useful with 32.64% responding that they were ‘Not at all useful’, and 20.37% selecting ‘A little useful’. A one-sample Wilcoxon test was used to test whether, for each online learning format the distribution of responses was significantly different from the midpoint of the scale. The distribution of responses was significantly different for both responses (W = -23175, P<0.0001 for ‘engaging’, W = -20730, P<0.0001 for ‘useful’.). These data indicate that, as a group, those participants who were taught using breakout rooms had a negative experience. Free text data revealed that participants found these awkward, and that other students were often reluctant to speak at all, making for an unsatisfactory learning environment.

**Fig 2:**
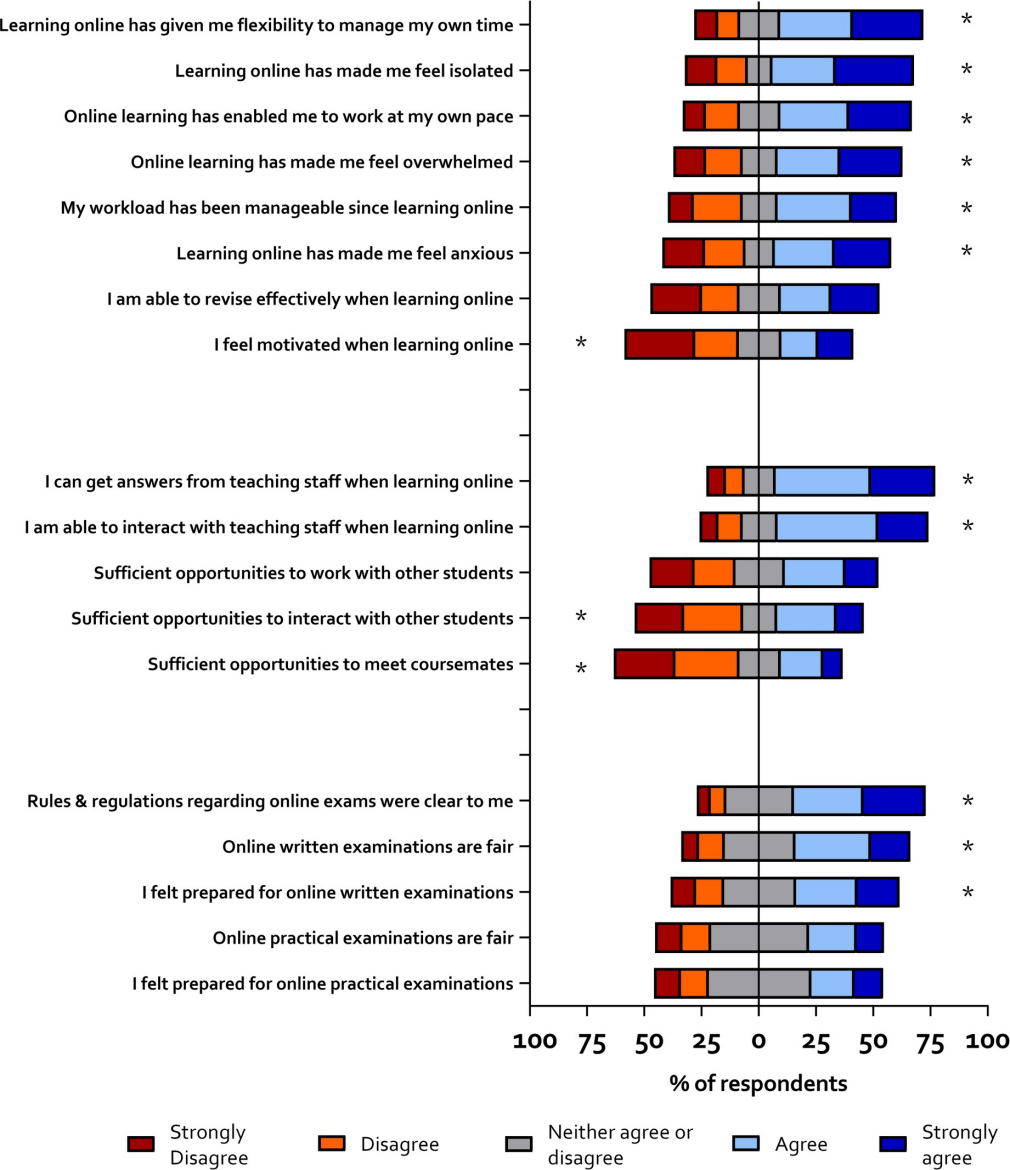
Student experience of different aspects of online learning. Distribution of responses was tested using a one sample Wilcoxon signed-rank test to determine whether they were significantly different from the midpoint of the range * = p<0.05. An asterisk to the right of the bar indicates the sample agreed with the statement, whereas an asterisk to the left means they disagreed. No asterisk means the sample neither agreed or disagreed.

Students were then asked to rate their agreement with a series of statements regarding their experience with Community and Collaboration, Workload, and Online Assessments. Again, students were largely positive about their experience, except for online practical classes, and regarding the opportunity to meet and interact with their peers. Summary results are shown in Fig 2, and the full dataset and analyses are shown in S5 File.

#### Community and collaboration

Thirty-seven percent of participants agreed that they had sufficient opportunities to interact with other students, whereas 46% percent disagreed. When asked if they would have liked more opportunities for interaction, 73% said yes. Over 40% of the sample agreed they had experienced sufficient opportunities to work with other students, and 18% disagreed. Sixty-one percent would have liked more opportunities to work with others. Only 25% felt they had enough opportunities to meet their coursemates, with 53% disagreeing with this statement. A large majority of participants agreed that they were able to get answers from teaching staff (69.8%) and interact with staff (66.0%) during online learning. Content analysis of qualitative data revealed that many participants felt there was no sense of community, and that group work was difficult to conduct online. The majority of free text comments suggested that teaching staff were not very responsive to e-mail and that it was harder to interact with staff and ask questions, though some found it easier to interact with staff online. This however confounds with the quantitative responses (see Fig 2), which suggested that students were able to interact effectively with staff.

#### My workload

Fifty-four percent of students responded that online learning made them feel overwhelmed, whereas 29% disagreed with this statement. Additionally, fifty percent said that online learning made them feel anxious, 61% felt isolated, and 48% did not feel motivated. These findings highlight the impact that online learning had on mental health. The advantages of online learning however were found in the flexibility that it offered. Fifty-seven percent said that it enabled them to work at their own pace, and 62% reported that it enabled them to manage their time flexibly.

#### Online assessments

Fifty percent of participants responded that online assessments were fair. The free text data (23% responses) suggested that Universities needed to make sure that the examination criteria were clear, but that participants largely wanted online assessments to continue. Participants also said online examinations were less stressful than in person exams, and open books exams were viewed positively. The possibility of internet issues during an exam was mentioned in 10% of comments, and 8% of comments were concerned with people cheating.

#### Accessing teaching and resources

**Fig 3:**
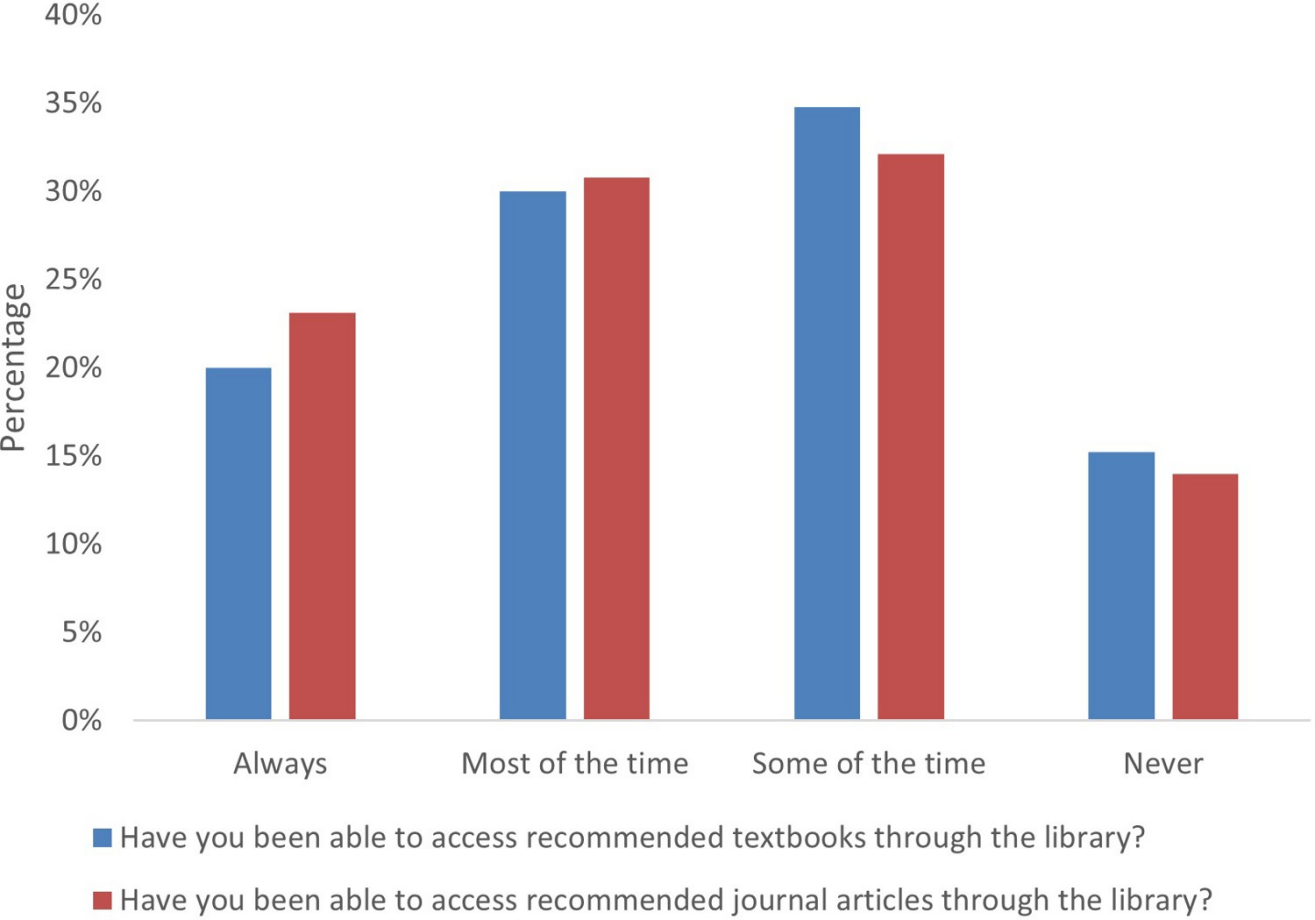
Student access to textbooks and journals via the library.

**Fig 4:**
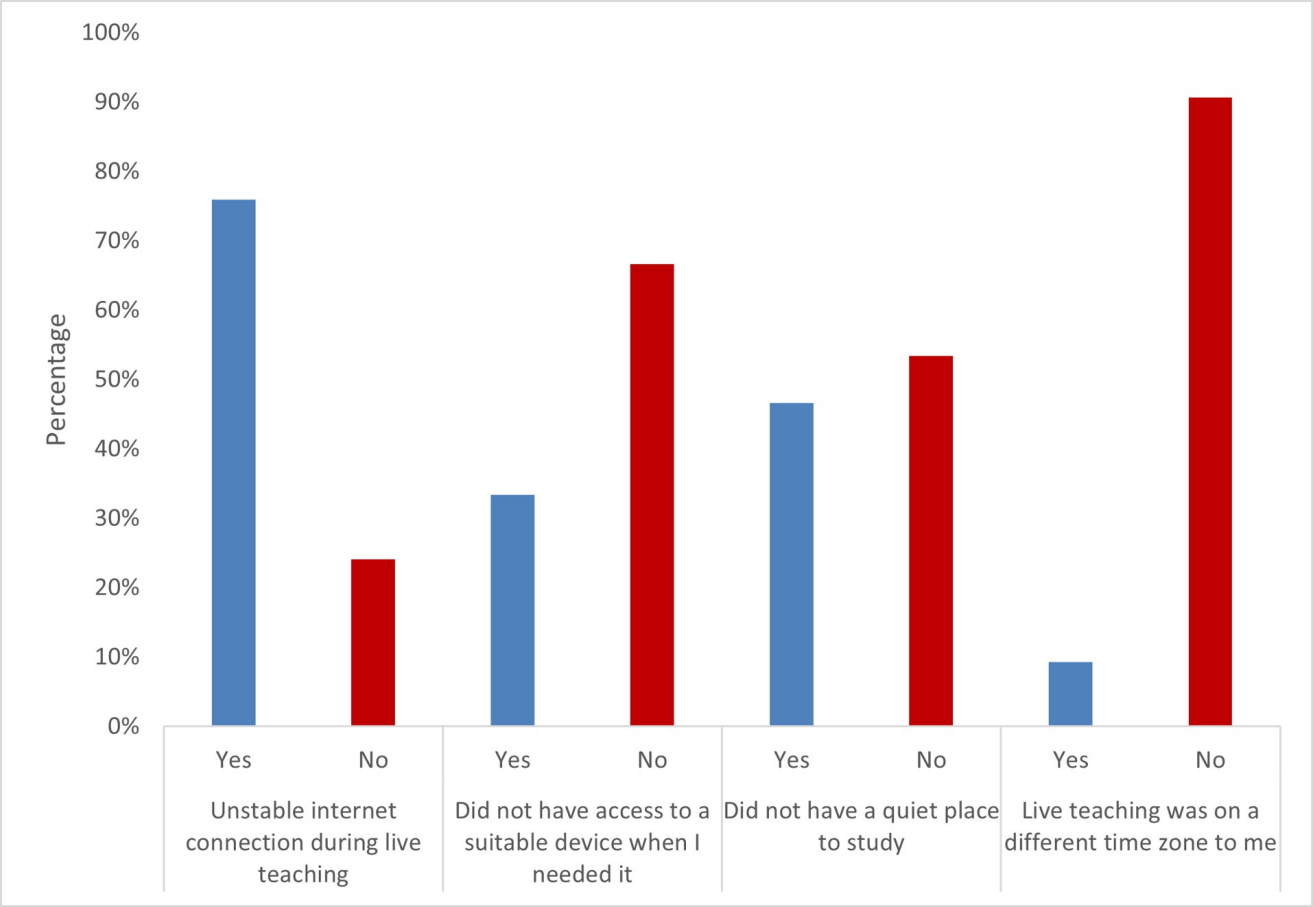
Issues with accessing online learning.

Over 75% of respondents experienced issues with internet connection during teaching, and just over a third of students were not able to access a device when they needed it. These findings highlight the need for recordings to be made available so that students are still able to access teaching. With regard to accessing recommended resources, participants had mixed experiences. Almost 70% of free text comments reported that they needed better access to resources. Some participants said that they were able to easily access online texts and journals, however others said that only physical copies of key textbooks existed, therefore they could not access them due to libraries being closed or living too far away. Some respondents highlighted that online resources were available, but that they did not always know how or where to access them, and that they needed training in the different VLEs. A number of participants praised the institutional library for the service and support they provided. Data are shown in Figs 3 and 4.

#### Managing my health when learning online

Qualitative data revealed that many participants felt more anxious, depressed isolated, overwhelmed, and lonely during online learning. Participants also reported that these issues impacted their ability to access learning, as they found it harder to engage and felt unmotivated. Content analysis found that over 76% of comments stated that their physical and or mental health was negatively affected by online learning. Participants reported physical health issues including back pain, eye strain, sedentary behaviours, and weight gain. Some respondents however, found that online learning helped them better manage their pre-existing health conditions, due to the flexibility of learning and lack of travel.

#### Caring for dependents

Twenty-four per cent of participants reported that they cared for dependents. Content analysis showed that 44% of comments reported online learning made caring for dependents harder, whereas 32% said it was easier. The impact that online learning had was mixed depending on individual circumstances. Participants with children often reported that online learning was harder, but some of these difficulties were due to school or childcare closures relating to the pandemic, rather than online learning per se. Participants with children found it challenging to find a quiet place to study, especially during live teaching, and found it more difficult to focus on their learning. Advantages of online learning for these participants was the lack of commute therefore being closer to a child’s school or dependent’s home; and the flexibility around when they studied due to recordings being available.

#### Thinking ahead

**Fig 5:**
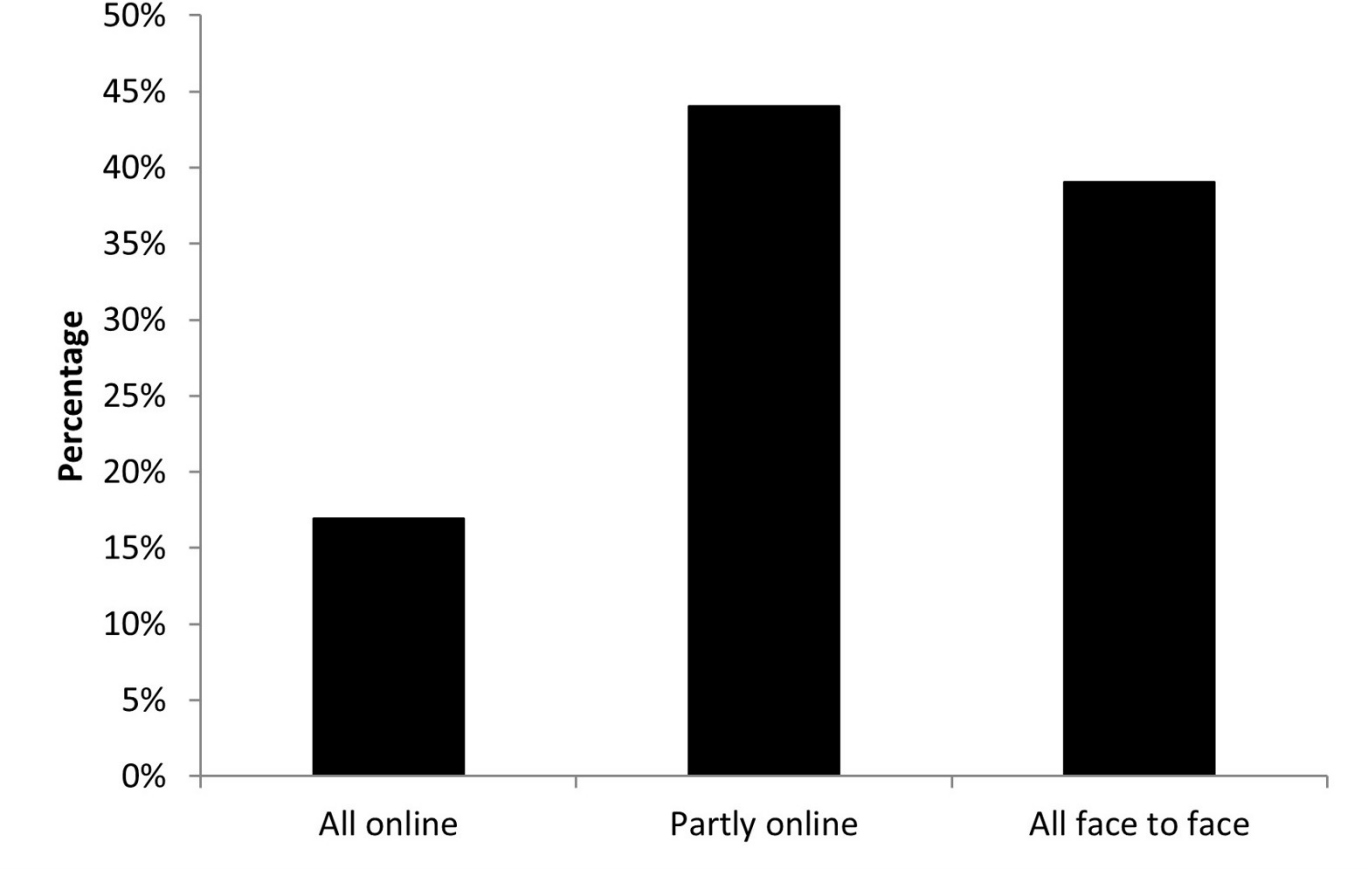
Participant preferences for future learning.

Participants were asked how they would like to learn in future (Fig 5), with 39% stating that they would like all face-to-face teaching, whereas 44% would like blended learning. Only 16% showed a preference for learning completely online.

## Discussion

This research study explored student perceptions of learning across Welsh HEIs during the pandemic in the 2021/22 academic year. The intention of the research was to identify sector-wide recommendations for future online learning practice, informed by student views.

### Principal findings

Qualitative analysis developed eight broad themes. These subsequently informed the development of the online questionnaire which was structured into six domains. The key findings from each domain are summarised below.

*Quality of online learning*: Overall, the majority of participants were satisfied with the quality of online teaching they received. However, students highlighted the need for additional interactive elements in both live and pre-recorded lectures. When asked about specific digital teaching techniques, breakout rooms were negatively perceived due to student feelings of awkwardness and limited engagement and interaction form other students, including non-use of cameras.

*Community and collaboration*: Overall, students felt that the sense of community was missing during online learning. Survey data reported that only 37% felt they had had sufficient opportunities to interact with other students during their learning and the overwhelming majority (73%) wanted more interaction. However, a large majority of participants (70%) agreed that they were able to get answers from teaching staff and interact with staff during online learning, suggesting that staff were able to interact with students during the pandemic period, and that perhaps peer-to-peer interaction was most missed.

*My workload*: Just over half of students surveyed (54%) indicated that online learning had made them feel overwhelmed. Additionally, half said that online learning made them feel anxious, isolated (61%), and un-motivated (48%). In contrast 62% indicated they benefitted from increased flexibility and (57%) the option to work at their own pace.

*Online assessments*: Half the students surveyed felt online assessments were fair, and that they largely wanted online assessments to continue, citing reduced stress. Concerns were raised regarding the clarity of assessment instruction, assessment criteria, and information on how to handle internet issues arising during examination periods.

*Accessing teaching and resources*: 75% of respondents experienced issues with internet connection during teaching, and just over a third of students were not able to access a device when they needed it.

*Managing my health when learning online*: Survey respondents free text data indicated many felt anxious, depressed isolated, overwhelmed, and lonely during online learning which impacted their ability to engage with digital learning content. Content analysis found that over 76% of respondents stated that their physical and/or mental health was negatively affected by online learning. Participants reported detrimental physical health aspects including back pain, eye strain, sedentary behaviours, and weight gain.

*Caring for dependents*: Almost a quarter *(24%)* of survey respondents had caring responsibilities. Content analysis showed that 44% of comments reported that online learning made caring for dependents harder. Participants with children found it challenging to find a quiet place to study, especially during live teaching, and found it more difficult to focus on their learning.

Further analysis was not undertaken on the basis of specific demographics, e.g. gender and individual higher education providers. This is partly due to the overall size of the sample, along with a desire to make general, sector-wide recommendations rather than recommendations for specific universities or groups of students. However future work could examine these questions in more detail

### Comparison to the literature

Key findings support previous research on remote learning which highlighted the challenges associated with navigating the new screen-based, remote, digital landscape [6]. These included limited peer and teacher interaction, [6] and issues with motivation and engagement [7]. Closure of institutional facilities such as libraries, study spaces and cafes [11] impacted on ability to access pre-recorded learning content and online assessments. Students struggled with single space living arrangements (such as university halls of residence) and unsuitable home environments [7], which often resulted in limited or no access to an appropriate study space. This also led to an unwillingness to switch cameras ‘on’ during live teaching sessions for fear of embarrassment. Technical challenges were further exacerbated access due to poor and intermittent Wi-Fi.

Similar to the current study, Bastos *et al* [6] surveyed Italian and Portuguese student perceptions of their transition to remote delivery of learning and teaching during the pandemic. Their study reported that students perceived learning was ‘not the same’ as prior classroom experiences due to a disconnect between students and staff, which impacted motivation. This loss of community and its resultant detrimental impact on students’ motivation to engage with their studies was in line with findings of the current study, where students discussed at length in the qualitive stage, their difficulties of learning, socialising and living in one space. Students reported a lack social connectedness and contact, and the loss of peer support and interaction. Engagement like this is a critical component of the learning environment (Bandura 1977).

Wu & Teets [7] reported a decrease in American chemistry students’ engagement and motivation to study at undergraduate level during the pandemic. Participants discussed their difficulties attending lecturers and watching pre-recorded materials, poor motivation, and issues surrounding access to required technology and safe/private study spaces. Authors concluded that “student’s home environment and circumstances play a significant role in their adaptation to emergency remote online instruction, with students who live in large family environments and/or are financial providers for their family facing the largest hurdles to maintaining their engagement.” (p3641). This effect is reported as more challenging in countries where the technological infrastructure for online learning was not as well developed before the pandemic [15]

Kulikowski et al. [30] highlighted the multi-dimensional impact of distance learning on cognitive demands, engagement, and the emotional experience of e-learning. Different domains had a differential impact on student perceptions of e-learning, not unlike the qualitative findings from the current study. Those students who reported difficulties in adapting to and navigating new technology, perceived their overall experience negatively. Whilst others, who found benefits and enjoyment from the increased flexibility in self-guided study perceived their experience to be useful and discussed their experiences in a positive way. A lack of information and dysfunctional communication at the university was reported by Kulikowski et al. (2021) which led to the perception of chaos. Whilst this was not the same for Welsh students, the impact of poor and disorganized communication had a significant impact on emotional experience, motivation and critically, the ability to study and meet the requirements of their courses on time. Kulikowski et al. (2021) also discussed the impact of digital immigrants teaching digital natives on ability to engage and study. This also resounded with the Welsh student experiences. Participants widely discussed staff lack of technical skills and ability to navigate the digital environment adequately, which was felt to further reduce (already diminished) learning opportunities.

Finally, authors drew attention to student perceptions at baseline, for e-learning and the impact on outcomes. Specifically, a positive attitude towards eLearning could enhance student engagement, motivation and ability.

Garris & Fleck [31] conducted a similar student survey to that of the stage 2 survey reported here. Their sample of 482 American undergraduate students evaluated their learning experiences during covid. However, their findings reported an overall negative evaluation of the transition to online learning. Specifically, courses were perceived to be less enjoyable, less interesting and of decreased pedagogic value. Similar to current findings, flexibility afforded by online learning was highlighted as a positive outcome. Authors identified that self-efficacy, emotional well-being and student engagement with online resources predicted positive evaluation. In line with current data that highlighted the importance of encouraging and supporting student engagement to ensure positive outcomes for learning.

The general level of satisfaction with online learning during the pandemic is perhaps surprising. Media coverage of Higher Education, in the UK, during lockdown, contained many negative reports of students who were unhappy with their university experience, particularly when this extended into a second academic year [32]. Some media outlets actively encouraged students to seek a refund on their tuition [33]. These reports were supported by petitions and other initiatives. For example a petition to the UK parliament, demanding refunds for students on their tuition fees, gathered over 270000 signatures, triggering a debate in parliament, although students were directed by the government to seek recourse through the relevant adjudicator [34, 35]. Our data suggest that a majority of students were generally content with most aspects of their educational experience, although this finding should be tempered with another finding that students were generally sympathetic to the efforts being made by their universities, in a time of crisis, and it seems reasonable to conclude that satisfaction levels would decrease if online learning were continued in the manner delivered during lockdown, particularly if elements such as mental health and poor WiFi were not addressed.

Overall, whilst the findings of the present study reported benefits of online learning in terms of flexibility and time management, institutions need to be aware of some of the downfalls of online learning, to be able to mitigate these. The present study suggested that students were largely satisfied with the quality of online learning during 2020–2021, suggesting that much content can be successfully transitioned to an online format. Nevertheless, whilst quality can be maintained with careful lecture design, qualitative data from both the focus groups and the survey suggested that students missed many aspects of the face-to-face learning experience. Furthermore, there were negative impacts on mental health including feeling overwhelmed, lack of motivation, and increases in depression and anxiety. Some of these can also be mitigated through effective pedagogical design, for example by using shorter formal lectures, with the time replaced by more informal discussion-based sessions [11].

### Recommendations for future practice

On the basis of the data in the present study, some key recommendations for future practice were developed by the team and then finalised by three authors (MB, AH, PN). The recommendations were focused on capturing lessons learning during the pandemic and turning them into positives aspects for online learning in a post-lockdown era. The recommendations were intended to be practically useful across the sector, but with a focus on the HE sector in Wales. They are categorised into three main areas: teaching practice, institutional level recommendations, and mental health and wellbeing.

### Teaching practice recommendations

- ***Use bite size lectures***. The structure of a lecture is key to maintaining motivation and engagement. Pre-recorded lectures should be broken into smaller sections (approximately 15 minutes), and live lectures should include breaks. The lecturer should ensure that their camera is switched on to foster a connection between the lecturer and the student. Closed captions should be used.
- ***Ensure asynchronous access***. Access to recorded content was a great success of lockdown teaching, and provides many obvious benefits to learning, some of which students now expect as basic requirements in return for tuition fees.
- ***Promote Interactivity and engagement***. Interactive activities such as quizzes polls, and live word clouds were popular features of online learning, as these encouraged interaction and kept students engaged. It is important that opportunities for students to ask questions are included. When mostly pre-recorded teaching is used, students valued the opportunity to attend live Question and Answer sessions to complement these.
- ***Set clear expectations for online breakout rooms*.** Breakout rooms can be made more effective by ensuring that students are given clear guidance in advance about what tasks they will be expected to do. Ideally, students should be allocated to the same group each time or be able to sign up to a particular room with students that they know. This helps reduce some of the awkwardness and allows students to develop social networks
- ***Practical classes should remain in-person wherever possible***. Practical classes did not translate well to an online medium. Some of the key learning can be achieved online, but the hands-on practical element is a key feature of in-person teaching.

### Institutional level recommendations

- ***Ensure consistent communication***. Students found it confusing when important information about their learning was communicated through different communication channels.
- ***Conduct a technology training needs analysis***. Many students reported that staff were not able to use the technology required for online learning, which hindered the learning experience. Similarly, many students themselves felt unequipped to use this technology. We recommend conducting a training needs analysis for both staff and students, to identify knowledge gaps.
- ***Ensure access to IT support***. One source of stress for students, particularly during online assessment periods, was when they had issues with technology but were unable to access help to resolve it.
- ***Address basic barriers to online learning***. Many students reported that they did not have access to an appropriate device for online learning, did not have a suitable place to study, and had issues with WiFi. Institutions should therefore ensure that students can access shared study spaces, computer laboratories, and that suitable equipment is available to loan. The technological requirements should also be made clear when students are making study choices.
- ***Foster a sense of community***. Many students reported that there were unique University experiences that they had missed due online learning during the pandemic. These consisted of rites of passage such as freshers’ week, the experience of campus based learning, meeting fellow students, and learning practical course skills. This should be factored in should students miss out on these events in future.

### Health and wellbeing recommendations

- ***Reduce student overwhelm*.** Many students reported that online learning was overwhelming. This can be reduced by providing clarity as to which components of a course are mandatory and which are optional and providing students with weekly checklists detailing which tasks need to completed.
- ***Provide first language mental health resources*.** Students that were first language Welsh speakers reported that they did not have access to mental health resources in Welsh. Welsh speakers reported that when talking about their mental health, it was more difficult conversing and expressing themselves in English.
- ***Schedule dedicated social opportunities at course or module level*.** Many students reported feeling isolated during online learning, therefore it is vital that social opportunities are timetabled to foster community.
- ***Timetabling breaks***. All learners need regular breaks, particularly when learning online–both *within* a teaching session and *between* teaching sessions. This ensures they can take comfort breaks, have a screen break for their eyes, move around, and consume food and drink.
- ***Be aware of social anxiety online*.** Enforcing that cameras are switched on during teaching may induce social anxiety. Some students feel that having cameras on is an invasion of privacy, increases stress and means they cannot move around. Furthermore, some students feel like they are being watched which can make them uncomfortable. Neurodivergent students are more likely to experience some of these difficulties. In the interests of inclusivity and accessibility, switching cameras on should not be mandatory.

## Supporting information

S1 File Full text of the part 2 survey. (PDF)

S2 File Participant demographics stage 1. Focus group participant demographics.
(DOCX)

S3 File Participant demographics stage 2. Survey participant demographics.
(DOCX)

S4 File Survey content analysis part 2. (DOCX)

S5 File Histogram of student experience. This is the raw data.
(CSV)
