# Supplementary material for: A pragmatic evaluation of university student experience of remote digital learning during the COVID-19 pandemic, focusing on lessons learned for future practice
Source: PLoS One. 2023 May 4;18(5):e0283742. doi: 10.1371/journal.pone.0283742 (PMC10159348; doi:10.1371/journal.pone.0283742)
Supplement: S1 File — (PDF) [file pone.0283742.s001.pdf]

Survey questions

About you - Demographics

Gender

Age

Year of study:

Degree programme: Undergraduate, postgraduate, foundation year

Please indicate which best reflects your general degree programme: (tick box)

- Medicine & dentistry
- Subjects allied to medicine
- Biological sciences
- Physical sciences
- Mathematics and computer sciences
- Engineering and technology
- Social sciences & Psychology
- Law and Business studies
- Languages
- Historical & philosophical studies
- Creative arts and design
- Education
- Other

When studying, where have you spent **the majority o**f your time living during the 2020/2021 academic year (i.e. September 2020 onwards): Halls, shared house/flat, at home with parents, living alone, home with partner/dependents (tickbox)

What is your home postcode (I.e. permanent residence, not term time accommodation):

Which countries have you resided in whilst studying (please state all):

Which university are you studying at?

This survey relates to teaching that has occurred from September 2020 onwards to the present day.

Quality of online teaching

On average, approximately how many hours of direct teaching content (i.e. live zoom lectures, pre-recorded, seminars, practicals etc) do you have each week (do not include self-directed learning such as course reading or revision).<br><br>Please Tick:					
0-4 hours	5-9 hours	9-13 hours	14 - 19 hours	20-24 hours	25 - 29 hours
30 – 34 hours	34 to 37 hours	38 hours+			

How satisfied were you with the ***quality*** of **live online lectures?**	Very dissatisfied	Dissatisfied	Neutral	Satisfied	Very satisfied	N/a<br><br>(I did not have live lectures)
How satisfied were you with the quality of the **pre-recorded lectures?**						
How satisfied were you with the						

quality of **live seminars/ tutorials**						
How satisfied were you with the quality of **online practical classes/virtual labs**						
How satisfied were you with the quality of **live Question & Answer sessions**						

We are interested to know how you think we could make online learning better:

**How could online lectures be made more effective? (free text)**
**How could pre-recorded lectures be made more effective?**
**How could live seminars/tutorials be made more effective? (free text)**
**How could online practical classes/virtual labs be made more effective? (free text)**

**How could live Question & Answer sessions be made more effective? (free text)**
Q: How engaging did you find Breakout rooms? (*If you did not experience breakout rooms then please go to next section)<br><br>(Not at all engaging, a little engaging, neutral, engaging, very engaging)<br><br>Q: How useful did you find breakout rooms?<br><br>(Not at all useful, a little useful, neutral, quite useful, very useful)<br><br>Q: How could breakout rooms be made more effective? (Free text)
Q: Does your course have practical elements to it? Yes No Don’t know<br><br>Q Have you had enough opportunities to develop those skills? Yes No Don’t know<br><br>Anything else you would like to add (Free text)
Q) Thinking about some of the different teaching methods you have received, was there anything particularly **effective** about how any of them were delivered?<br><br>A) Free text response
Q) Thinking about some of the different teaching methods you have received, was there anything particularly **ineffective** about how any of them were delivered?<br><br>A) Free text response
Q) Did you experience any other teaching methods that have not already been mentioned? (If no, leave blank)<br><br>A) Free text response
Q) During the academic year of Sept 2020 to 2021, was any of your learning cancelled and not subsequently re-arranged?<br><br>Yes No Not sure

Q) Is there anything you would like to add about this? (Free text)
Q) Were you given a timetable of when teaching materials would be released?<br><br>Yes No Not sure Not applicable<br><br>Q) Is there anything you would like to add about this? (Free text)
Q) Were teaching materials (e.g. pre-recorded lectures) made available on time?<br><br>Never rarely some of the time most of the time always

Community and collaboration

Q) I have had sufficient opportunities to interact with other students whilst learning online<br><br>(Strongly disagree, disagree, neither agree nor disagree, agree, strongly agree)<br><br>Q) Would you have liked to have had more opportunities to interact with other students<br><br>A) Yes/No /Don't know
Q) I have had sufficient opportunities to work with other students whilst learning online (e.g. group presentations, group projects, discussions)<br><br>(Strongly disagree, disagree, neither agree nor disagree, agree, strongly agree)<br><br>A) Yes No Don't know

Would you have liked to have had more opportunities to work with other students?<br><br>Yes no don’t know<br><br>Free text – any other comments?
Q) I have had sufficient opportunities to meet new coursemates (either online or face to face)?<br><br>(Strongly disagree, disagree, neither agree nor disagree, agree, strongly agree)<br><br>Would you have liked to have had more opportunities?<br><br>Yes No Don’t know
Q) I am able to interact with teaching staff when learning online<br><br>(Strongly disagree, disagree, neither agree nor disagree, agree, strongly agree)<br><br>Q) I can get answers to my questions from teaching staff when learning online<br><br>(Strongly disagree, disagree, neither agree nor disagree, agree, strongly agree)<br><br>Anything else you would like to add about interacting with teaching staff?<br><br>(Free text)

My workload

Q) My workload has been manageable since learning online<br><br>(Strongly disagree, disagree, neither agree nor disagree, agree, strongly agree)

Q) Online learning has made me feel overwhelmed

(strongly disagree, disagree, neither agree nor disagree, agree, strongly agree)

Q) Online learning has enabled me to work at my own pace

(strongly disagree, disagree, neither agree nor disagree, agree, strongly agree)

Q) Learning online has made me feel anxious

(strongly disagree, disagree, neither agree nor disagree, agree, strongly agree)

Q) Learning online has given me flexibility to manage me own time

(strongly disagree, disagree, neither agree nor disagree, agree, strongly agree)

Q) Learning online has made me feel isolated

(Strongly disagree, disagree, neither agree nor disagree, agree, strongly agree)

Q) I feel motivated when learning online

(Strongly disagree, disagree, neither agree nor disagree, agree, strongly agree)

Q) I am able to revise effectively when learning online

(Strongly disagree, disagree, neither agree nor disagree, agree, strongly agree)

Online Assessments

Q) Online written examinations are fair

(Strongly disagree, disagree, neither agree nor disagree, agree, strongly agree)

Q) Online practical examinations are fair

(Strongly disagree, disagree, neither agree nor disagree, agree, strongly agree)

Q) I felt prepared for online written examinations

(Strongly disagree, disagree, neither agree nor disagree, agree, strongly agree)

Q) I felt prepared for online practical examinations

(Strongly disagree, disagree, neither agree nor disagree, agree, strongly agree)

Q) The rules and regulations regarding online exams were clear to me

(strongly disagree, disagree, neither agree nor disagree, agree, strongly agree)

What advice would you give to your University about online examinations? (Free text)

Accessing teaching and resources

Q) Have you been able to access recommended textbooks through the library?

Always, most of the time, some of the time, never

Q) Have you been able to access recommended journal articles through the library

Always, most of the time, some of the time, never

What advice would you give to your University about accessing resources?

(Free text)
**Did you experience any of the following whilst learning online?**
Unstable internet connection during live teaching Yes/no<br><br>Did not have access to a suitable device when I needed it Yes/no<br><br>Did not have a quiet place to study Yes/no<br><br>Live teaching was on a different time zone to me Yes/no

Managing my health when learning online

We are providing guidance to Universities to help them find the best ways to support their students, by asking whether your health (mental or physical) plays a role in your ability to study online. For example, some people may find online teaching easier or harder to access due to their health conditions.

Q) Has any aspect of your physical or mental health been positively or negatively impacted by learning online? (free text)<br><br>Q) Has any aspect your physical or mental health positively or negatively affected your ability to **access or engage** with online learning? (free text)

Caring for dependents

Q) Do you provide care for any dependents (i.e. this does not include paid work where care is part of your employment)? Yes No<br><br>Q) If yes, how has learning online affected your ability to care for dependants?<br><br>(Free text)

Thinking ahead…

Q) In future, I would like to learn:

(All online, partly online, all face to face to face) Tick box

Q) Any other comments about future learning

(Free text)

Q) **Is there anything you would like to add about your experience of online learning?**

Free text

Q) **If you were to make one recommendation to your university for effective online learning, what would you say?**

Free text
