# Supplementary material for: A pragmatic evaluation of university student experience of remote digital learning during the COVID-19 pandemic, focusing on lessons learned for future practice
Source: PLoS One. 2023 May 4;18(5):e0283742. doi: 10.1371/journal.pone.0283742 (PMC10159348; doi:10.1371/journal.pone.0283742)
Supplement: S2 File — Focus group participant demographics. (DOCX) [file pone.0283742.s002.docx]

|  | **Participants** | **N (%)** |
| --- | --- | --- |
| Participants |  | 54 |
| Gender |  |  |
|  | Female | 32 (59) |
|  | Male | 21 (39) |
|  | Non-binary | 1 (2) |
| University |  |  |
|  | Swansea University | 22 (41) |
|  | Aberystwyth | 17 (31) |
|  | Cardiff University | 4 (7) |
|  | Cardiff Met | 3 (5) |
|  | University of Wales Trinity Saint David | 5 (9) |
|  | Bangor University | 3 (5) |
|  |  |  |
| Year of study |  |  |
|  | Foundation Year | 1 (2) |
|  | Masters | 3 (5) |
|  | PhD | 1 (2) |
|  | Undergraduate 1^st^ Year | 25 (46) |
|  | Undergraduate 2^nd^ Year | 15 (28) |
|  | Undergraduate 3^rd^ Year | 4 (7) |
|  | Undergraduate 4^th^ Year | 1 (2) |
|  | Not recorded | 4 (7) |

**Participant Demographics for Stage 1 (Qualitative)**
