# Supplementary material for: A pragmatic evaluation of university student experience of remote digital learning during the COVID-19 pandemic, focusing on lessons learned for future practice
Source: PLoS One. 2023 May 4;18(5):e0283742. doi: 10.1371/journal.pone.0283742 (PMC10159348; doi:10.1371/journal.pone.0283742)
Supplement: S3 File — Survey participant demographics. (DOCX) [file pone.0283742.s003.docx]

**Stage 2 Survey**

**Participant demographics**

|  | | N | % |
| --- | --- | --- | --- |
| Gender | Female | 466 | 69.04% |
|  | Male | 188 | 27.85% |
|  | Non-binary | 13 | 1.93% |
|  | Prefer not to say | 8 | 1.19% |
| Year of study | Foundation year | 34 | 5.04% |
|  | Year 1 | 229 | 33.93% |
|  | Year 2 | 217 | 32.15% |
|  | Year 3 | 132 | 19.56% |
|  | Year 4 | 36 | 5.33% |
|  | Year 5 | 12 | 1.78% |
|  | Year 6 | 15 | 2.22% |
| Degree subject | Medicine & dentistry | 40 | 5.93% |
|  | Subject allied to medicine | 63 | 9.33% |
|  | Biological sciences | 72 | 10.67% |
|  | Physical sciences | 28 | 4.15% |
|  | Mathematics and computer sciences | 32 | 4.74% |
|  | Engineering and technology | 30 | 4.44% |
|  | Social sciences & Psychology | 102 | 15.11% |
|  | Law, Business Studies and Marketing | 102 | 15.11% |
|  | Languages | 17 | 2.52% |
|  | Historical and philosophical studies | 46 | 6.81% |
|  | Creative arts and design | 69 | 10.22% |
|  | Education | 74 | 10.96% |
