# Supplementary material for: A pragmatic evaluation of university student experience of remote digital learning during the COVID-19 pandemic, focusing on lessons learned for future practice
Source: PLoS One. 2023 May 4;18(5):e0283742. doi: 10.1371/journal.pone.0283742 (PMC10159348; doi:10.1371/journal.pone.0283742)
Supplement: S4 File — (DOCX) [file pone.0283742.s004.docx]

**Part 2 Survey – content analysis of free text questions**

The table below shows the three most commonly occurring themes for each question.

| Q14. How could online lectures be made more effective?   - Technical support - Clear and efficient timetabling - Cannot improve (positive - I.e. already good enough) |
| --- |
| Q15. How could pre-records be made more effective?   - Shorter chunks - Add tasks and activities - Unspecific (positive) |
| Q16. How could live seminars/tutorials be made more effective?   - More interactive components - Mandatory attendance &interaction - Specific focus |
| Q17. How could online practical classes be made more effective?   - Can’t make better – need to do in person (i.e. they don’t work) - Make more interactive - Technology |
| Q18 How could live Q&As be made more effective?   - Pre submit questions - No improvements needed - Encourage participation from other students |
| Q21. How could breakout rooms be made more effective?   - Structured - Dislike them - Pre agreed groups |
| Q. 24 Anything else you would like to add about practicals when learning online?   - Negative - Essential - In person |
| Q25. Thinking about some of the different teaching methods you have received, was there anything particularly effective about how any of them were delivered?   - Recordings - Passion and staff skill - In person/live |
| Q37. Any other comments about online learning   - No community - Group work negative online - Want face to face* - Not enough collaboration* - Group work negative general* |
| Q42. Anything else you would like to add about interacting with teaching staff?   - Lecturers were not responsive to email - Harder to interact/ask questions online - Easier/can interact online |
| Q45. What advice would you give to your University about online examinations?   - Make sure criteria are clear - Keep doing them - Allow sufficient time |
| Q47. What advice would you give to your University about accessing resources   - Need better access - Good access - Good service |
| Q50. Has any aspect of your physical or mental health been positively or negatively impacted by learning online?   - Negative impact - Positive impact - Mixed impact |
| Q51. Has any aspect of your physical or mental health positively or negatively affected your ability to access or engage with online learning?   - No impact - Yes it had an impact - Motivation harder |
| Q53. If yes, how has learning online affected your ability to care for dependents?   - Harder - Easier - Harder due to homeschool |
| Q55. Any other comments about future learning   - Want in person - Want blended - Remain online |
| Q56. Is there anything you would like to add about your experience of online learning?   - Don’t like it - Like it - Mixed |
| Q57. If you were to make one recommendation to your university for effective online learning, what would you say?   - Face to face - Interactive - More support |

*indicates equal number of responses
